# Behavioral responses to artificial insemination and the effect of positive reinforcement training

**DOI:** 10.1371/journal.pone.0310537

**Published:** 2024-10-10

**Authors:** Jennifer L. Heinsius, Marina A. G. von Keyserlingk, Daniel M. Weary

**Affiliations:** Animal Welfare Program, Faculty of Land and Food Systems, The University of British Columbia, Vancouver, BC, Canada; University of Florida, UNITED STATES OF AMERICA

## Abstract

Dairy cattle experience a variety of stressors associated with routine farm practices, including injections, pen movements, regrouping, and artificial insemination. The primary objective of this study was to assess ear position (categorized according to 5 positions) and body movement responses of heifers during their first experience of artificial insemination, in comparison with responses during baseline observations before and after this procedure. A secondary objective was to test whether heifers exposed to positive reinforcement training (PRT) responded differently to this procedure. We tested 12 heifers (13 ± 0.7 mo. old); 7 were trained using PRT (as part of another study) and 5 had no experience with PRT. Ear axial and ear forward positions were more frequent during observations before and after artificial insemination relative to during artificial insemination (4.57 ± 0.82 vs. 0.31 ± 0.82 times/period, and 3.45 ± 0.51 vs. 0.50 ± 0.51 times/period, respectively). Backward pinned ear position was observed less frequently before and after versus during artificial insemination (0.12 ± 0.76 vs. 3.76 ± 0.76 times/period). We found no effect of period relative to artificial insemination on any of the 4 body movements assessed (leaning forward, backward, and steps taken with front legs and back legs).We found an effect of PRT on forward ear position (1.00 ± 0.51 times/PRT group vs. 2.95 ± 0.60 times/control group), and found that control heifers leaned forward and took more steps with their front legs than did PRT heifers (0.93 ± 0.57 times/PRT group vs. 3.55 ± 0.67 times/control group and 1.29 ± 0.68 times/PRT group vs. 3.85 ± 0.81 times/control group). We conclude that heifers experiencing artificial insemination for the first time express distinct ear positions that are consistent with responses to a negative emotional state. Further work is required to validate these responses and to determine the extent that they can be used to assess affective responses to this and other procedures.

## Introduction

Dairy cattle may experience unpleasant procedures including injections, ultrasound examination, and artificial insemination. In each case, the procedure itself and the associated restraint and handling before, during and after the event, can be aversive depending on the novelty and how the procedure is performed [1–3]. How these procedures are performed may be driven by the desire for worker efficiency or to reduce the risk of injury to animal handlers [4]. Some literature has focused on refinements to procedures such as disbudding [5, 6] and recent work and reviews have sometimes included an explicit focus on the impact of these procedures on affective states in farm animals [7–9].

Research investigating the effects of management procedures on affective state often relies upon physiological measures of the hypothalamic-pituitary-adrenal axis and the sympathetic nervous system, including cortisol, heart rate, and eye temperature [10–12], but collecting samples needed for some measures (e.g. blood collection) can also be a stressor and thus affect results [13]. Behavioral indicators of affective states include play, escape attempts, and changes in movement patterns and body postures [14–16]. Ear position has been suggested as an indicator of emotional state in a variety of species (sheep: [17]; pigs: [18]; horses: [19]), and work on cattle suggest certain ear positions are associated with positive or neutral states [20].

Positive reinforcement training (PRT) can increase voluntary participation [21, 22], even when subjecting animals to unpleasant procedures [23]. PRT allows the animal to control when, and how they wish to proceed [24]. The use of PRT may also improve the safety of human handlers; for example, Carlstead et al. [25] reported that captive elephants that bonded to handlers (due in part to positive interactions) were safer to use and showed less aggression and apprehension compared with those that had no positive interactions with handlers.

Cattle handling methods are often based on using the animal’s ‘flight zone’ to direct movement [26], and as such are inherently fear based [27]. The development of PRT has been driven in part by zoo handlers who desired methods that are not fear based [23, 28], and are thus believed to improve animal welfare and relationships between zookeepers and the animals [25]. Recent evidence from our group suggests that PRT may provide benefits for dairy cattle exposed to otherwise aversive situations [29, 30].

The primary aim of the current study was to describe how ear position and movement parameters in heifers vary in response to exposure to artificial insemination, a management procedure that is assumed to be aversive [31]. A secondary aim was to assess if animals trained using PRT respond differently to this procedure. We hypothesized that naïve heifers would exhibit changes in ear position and body movements during artificial insemination relative to baseline observations before and after the procedure. The previous literature on ear positions in cattle was not sufficiently developed for us to make specific predictions regarding which behaviors would change. We also hypothesized that heifers previously trained using PRT would experience reduced fear relative to heifers that received no training, and thus expected that their behavioral response to the procedure would be mitigated relative to control heifers that did not receive training.

## Materials and methods

### Animals and housing

This study was conducted from February to May of 2022 at The University of British Columbia (UBC) Dairy Education and Research Centre located in Agassiz, Canada. All procedures were approved by The University of British Columbia Animal Care Committee (UBC Animal Care Committee protocols A18-0174-A003). We observed 12 dairy Holstein heifers averaging ± SD 13.6 ± 0.7 mo of age when first subjected to artificial insemination (min age:12.6 mo, max age: 14.9 mo; age varied depending on breeding decisions by the barn staff). Heifers were moved from the heifer barn to the main barn separately. Regrouping occurred at least 2 days before artificial insemination. All heifers were housed in a freestall barn, in a pen that held a maximum of 12 animals (including at least some others on the same study).

All 12 test heifers had participated in a previous study on PRT as described by Heinsius et al. [30]. During that study, at approximately 5.0 ± 0.6 mo. of age, PRT heifers participated in 6 wks of daily target training for 5 min to enter a chute and remain there for 1 min while being given food reinforcement; control heifers were moved and restrained for 1 min using the same chute for 6 wks using routine handling methods but were given no food reinforcement. In the months that followed some animals were sold, such that at the onset of the current study a total 12 of these heifers were available, 7 of which had received PRT treatment (averaging ± SD 13.7 ± 0.7 mo old), and 5 that had been assigned to the control condition (averaging 13.3 ± 0.7 mo old).

The sample size required was estimated for the primary aim (i.e. response during artificial insemination versus baseline) for our primary response variable (ear position). Proctor and Carder (2014) found cows spent on average, 1.95 min with their ears held backwards (i.e. ‘ear-position 3’) while being stroked, versus just 0.87 min in this position during observations when they were not stroked, with a SD of 1.15 [20]. Based upon these values and using the Power procedure in SAS Studio (for a one-sample means t-test; specifying power = 0.8 and α = 0.05), we estimated that a sample of 11 heifers was required.

### Procedures

When heifers approached breeding age they were moved from a heifer barn to the main barn: this occurred at different times for each heifer, but in every case at least 2 days before artificial insemination. As part of routine farm practice, farm staff observed heifers daily for behavioral signs of estrus and by using collar-mounted automated activity monitors. Within 12 h of being identified in estrus, heifers were moved by staff from their home pen to the adjacent breeding pen for artificial insemination and for the associated observations described below. Heifers were then moved back to the home pen after spending a total of approximately 20 min in the breeding pen.

When first brought into the breeding pen, heifers were gently guided into a headlock (the same location where artificial insemination would later be performed). Heifers were then observed during 3 periods, each 3 min in length: before, during and after artificial insemination. The before period began immediately after the heifer was first restrained in the headlock. After this observational period, heifers were kept in the headlock for another 7 min, after which they were bred using artificial insemination by experienced farm staff; the during artificial insemination observational period began at the onset of the procedure. The average time (± SD) to complete artificial insemination was 2.5 (± 1.5) min; but responses were measured during the first 3 min following the onset of the procedure regardless of the length of the procedure. Immediately following the procedure (or the 3 min observation period, whichever was longer), heifers were returned to their home pen. On the following day (at the same time of day), heifers were moved back to the breeding pen and again restrained in the headlock for the final (after artificial insemination) observational period. The 12 test heifers were each bred on a different day, all between February and May 2022.

The breeding pen was adjacent to the home pen, such that heifers had visual and auditory contact with pen mates in the home pen. The breeding pen contained 6 headlocks and measured 3.2 x 4.9 m. During observations, only three people were present: the trainer and a second observer (both located outside the pen facing the headlock), and a member of the farm staff who performed the artificial insemination.

During all three observational periods PRT heifers participated in target training, with the trainer standing close to the head of the heifer and presenting the target as soon as she entered the headlock. Each individual heifer had only one trainer, but over the course of the study 3 different trainers were used. All trainers were trained by the same person and their training was assessed before they conducted any training on their own. We used a red cardboard target, as described by Heinsius et al. [30], that was already familiar to these trained heifers. The target was first presented in front of the headlock and heifers were allowed 10 s to touch it with their nose. If they did not nose touch the target, the target was removed. This procedure was then repeated every 10 s for 3 min. A grain reward (~50 g) was provided each time the heifer touched the target with her nose. Every PRT heifer successfully participated in the target training during the before artificial insemination observational period (with an average proportion of successful target touches = 0.88 ± 0.15, min = 0.67, max = 1.00). For heifers in the control group, the trainer stood quietly, in the same location as with PRT heifers but remained still and quiet throughout the 3 min observational period.

### Behavior recording

**Fig 1:**
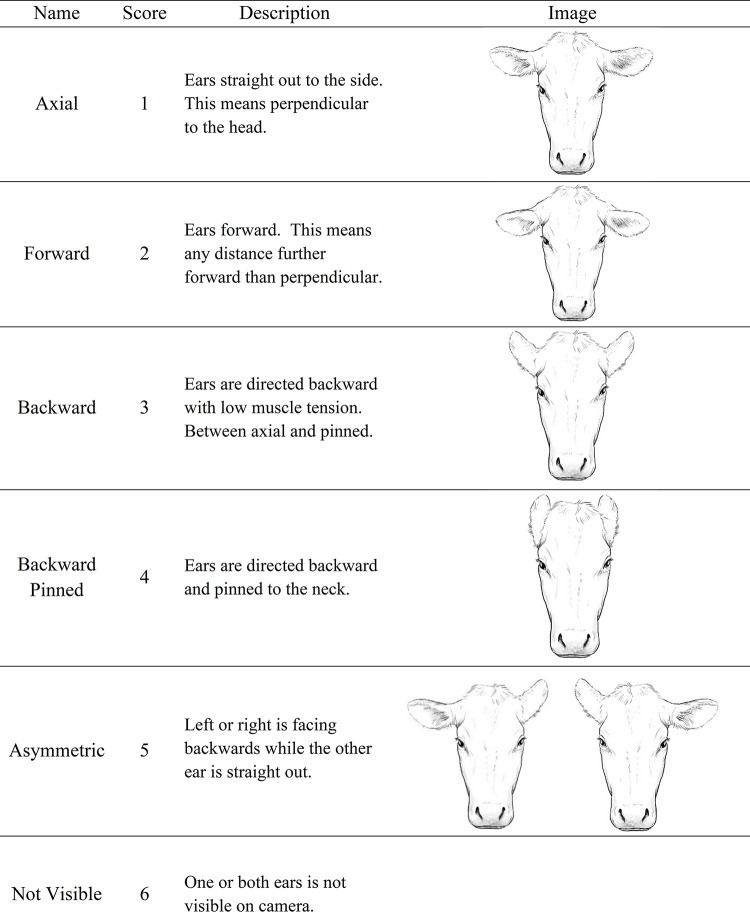
Description and artistic representation of the ear positions observed during artificial insemination (adapted from de Oliveira and Keeling 2018).

We recorded ear positions and body movements during the 3-min observational periods before, during and after the artificial insemination. We used a camcorder (Canon Vixia HF R82, Ota City, Tokyo, Japan); mounted on a tripod positioned 1 m in front of the heifer, to record ear position. Ear positions was categorized as defined in Fig 1 (as adapted from de Oliveira and Keeling [14] and recorded using instantaneous scan sampling every 5 s during each 3-min period. Scans were totaled for analysis.

A GoPro camera (Hero 4, GoPro Inc., USA; mounted on the fence 2 m behind the heifer), was used to record body movements, including the number of ‘steps’ (i.e. either leg lifts and full steps) taken with the front legs and with the rear legs, and the number of times the heifer leaned forward (defined as when the shoulders hit the headlock) or backward (the ears pulling against the headlock). Each movement was recorded as a count and counts were totaled over the observational period for analysis.

Both observers watched ten training videos and scored all behaviors and ear positions. An initial interobserver-reliability assessment was performed and differences were discussed to improve scoring criteria. A week later the same observers watched a new set of ten videos (to calculate the inter-observer reliability which varied from 0.64–0.98), and these same videos were scored again a week later (to calculate intra-observer reliability, which varied from 0.66–0.98). Two ear scores (asymmetric and not visible) had lower reliability, due to inconsistencies in scoring partially visible ears, and hence are not reported further. Once training and reliability assessments were complete, one observer scored all the videos for the behavioral measures and the second observer scored all videos for ear position. Neither observer was blind to treatment (as target training could be observed on the video), but both observers were unaware of the predictions.

### Statistical analysis

We used a linear mixed model (using PROC Mixed in SAS Studio), specifying individual cow ID as a random effect, and the effect of treatment period as a within-subject effect. We expected that responses pre- and post-artificial insemination would be similar, so the first step to our analysis was to compare these two periods using a linear mixed model. No difference was found for any outcome measure, so we used a second model to compare the combined baseline (averaging pre- and post-observational periods) with the response during the artificial insemination. We also included in the model the effect of PRT (tested as a between-subject effect), and the interaction between PRT and period. All data and code are available in the supplementary materials (doi.org/10.5683/SP3/T4LOS3).

## Results

**Fig 2:**
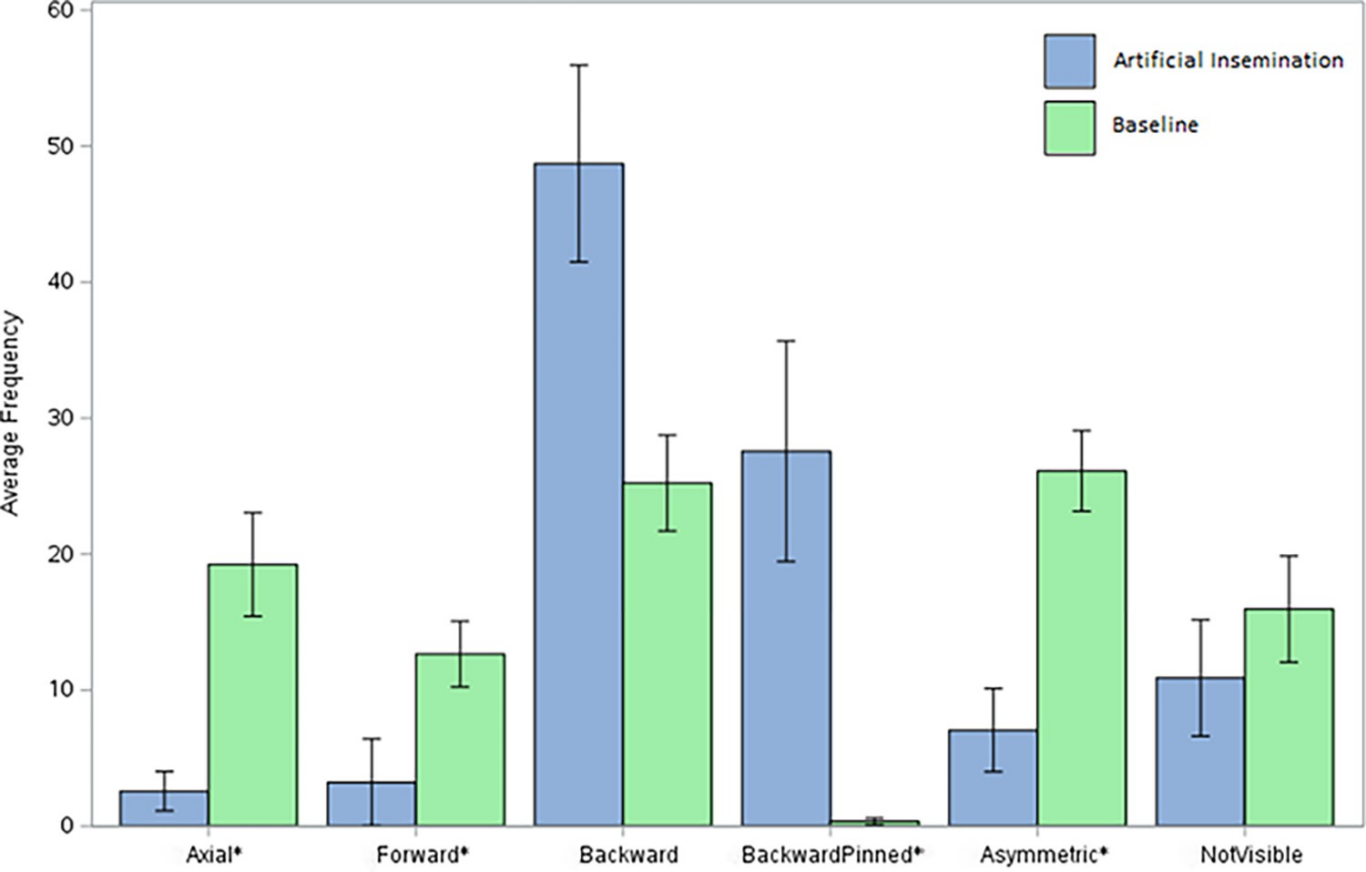
Average frequency of ear position relative to artificial insemination. Bars illustrate the mean ± SE number of times that heifers (n = 12) expressed each of five different ear positions (and when ears were unable to be viewed). Ear positions were scored at 5 s intervals for 3 min during artificial insemination (blue bars) and during baseline periods (also 3 min each) pre- and post-artificial insemination. Responses did not differ between pre- and post- periods, so these were averaged (green bars).

Ear postures differed during artificial insemination versus during baseline observations (Fig 2). Backward pinned ear positions were more common during artificial insemination than during the baseline, and axial, forward, and asymmetric ear positions were more common during the baseline. We found no effect of observational period for any of the movement parameters recorded (see S1 Table in the supplementary materials for full data).

We detected no effect of PRT for 4 of the 5 ear positions (Table 1), but the forward ear position was observed approximately 3 times more frequently in Control vs. PRT heifers. Control heifers were observed moving forward more than three times more frequently than PRT heifers, as well as taking more steps with their front legs. We found no other effect of PRT on any of the other response measures, and no interaction between period relative to artificial insemination and treatment for any of the response measures (see S2 Table in the supplementary materials for full data).

**Table 1:** Mean ± SE number of times, during a 3-min observational period, heifers were observed showing each of five ear positions (and when ear position could not be scored), as well as four body movements.

		PRT	Control	F _[__1__,__10__]_	P
**Ear Positions**	Axial	2.79 ± 1.02	2.10 ± 1.20	0.35	0.57
	Forward	1.00 ± 0.51	2.95 ± 0.60	6.14	0.03
	Backward	6.93 ± 1.00	5.30 ± 1.18	1.11	0.31
	Backward Pinned	1.43 ± 0.71	2.45 ± 0.85	0.85	0.38
	Asymmetric	3.46 ± 0.65	4.15 ± 0.77	0.47	0.51
	Not Visible	2.89 ± 0.88	2.05 ± 1.05	0.38	0.55
**Movement Parameters**	Forward	0.92 ± 0.56	3.55 ± 0.67	8.94	0.01
	Backward	0.39 ± 0.37	1.50 ± 0.44	3.64	0.09
	Front Steps	1.29 ± 0.68	3.85 ± 0.81	5.88	0.04
	Back Steps	12.25 ± 2.66	12.45 ± 3.14	0.00	0.96

Results are shown separately for heifers that had received PRT (*n* = 7) and Control heifers that had not been trained (*n* = 5). Also shown is the F-statistic for the within-subject effect of PRT, and the corresponding p-value.

## Discussion

Ear positions of naïve heifers changed when first experiencing artificial insemination, a procedure we assumed to be aversive to these animals [31]. During baseline observations (before and after the procedure), we saw higher frequencies of forward, axial, backwards, and asymmetric ear positions. A study by de Oliveira and Keeling reported that axial and backwards ears were the most observed when cattle were queuing, brushing, or feeding, all relatively low arousal activities [14]. During artificial insemination, the heifers in the current study used backwards and backwards pinned ear positions; backward pinned was a position rarely observed during de Oliveira and Keeling’s study [15]. It is worth noting that we observed the backwards ear position during both artificial insemination and non-artificial insemination periods. We suggest that the distinction between backwards and backwards pinned is important, and that the backwards pinned position is more closely linked to aversive events. A study focusing on pain in cows classified ear position in two ways: backwards ear and “low/lamb ears” and suggested that the presence of muscle tension in the ear was an indicator of pain [32]. This is the primary difference between the backwards ear position and the backwards pinned position. Future work should focus on validating how specific ear positions relate to level of arousal and affective valence. Based on our results, we suggest that ear position may be a useful behavioral indicator of cattle responses to aversive procedures.

We found no effect of artificial insemination on the gross movements parameters we measured. Movements in restrained animals are difficult to study, in part because restraint itself can cause distress [33]. Restraint can also limit the types of gross motor responses that an animal can express [34]. To overcome these limitations, we suggest that future studies use animals that are not restrained, or conversely that researchers do not use these measures when animals are physically restrained.

Our study was designed around our primary objective (i.e. detecting the within-subject effect of observational period). Our secondary aim was to test the hypothesis that PRT mitigates responses to insemination, but our between-subject test of this was less powerful, perhaps explaining our failure to detect differences. Acknowledging this limitation, we see no evidence that PRT (as applied in the current study) was effective at mitigating responses to artificial insemination. Previous studies have suggested training for at least 3 d before using the training during a procedure [35]. In the current study, heifers were familiar with target training as part of their experience in a previous setting, but they had never used target training in this new environment, or when socially isolated. The use of target training in the current study built upon our previous work for which this method was well suited. However, future work could benefit from the use of simpler methods to reduce fear during novel procedures. Research has shown that habituation to location and human contact can reduce fear [36, 37]; future studies could train heifers for voluntary participation in leaving the home pen as well as entering the headlocks before the novel aversive event.

We found that heifers immediately participated in target training during baseline events, but noted reduced participation during artificial insemination suggesting that the target task was insufficient to distract the heifer during this procedure. We also noted that, during the artificial insemination procedure, some heifers would touch the target but not consume the food reward, again suggesting that the PRT was not sufficiently distracting. Research has shown that pairing aversive events with food reward can lead to extinction of the reinforcing effect of the food by reducing motivation to approach [38]. However, other research suggests that food reward can be used to initiate fear extinction when used appropriately, suggesting that target training could be used in more intentional ways to reduce the aversiveness in future work [39].

The parameters we used in this study were likely well suited to detect acute affective experiences, but we also encourage future studies to look at the effect of artificial insemination on longer-term affective states and mood [40]. Our results suggest that ear position can be used to detect the acute effects of procedures on the emotional state of heifers, but this method may be less useful for understanding the duration of any effects [41].

## Conclusions

Ear positions changed when naïve heifers experienced artificial insemination; the changes we observed are consistent with a negative affective response to this procedure. We found no evidence that PRT was able to mitigate these changes in ear position in response to artificial insemination, but our design was not powered to test this effect.

## Supporting information

S1 Table LS mean ± SE counts per observational period for each ear position and body movement assessed. Naïve heifers were observed for 3 min during the artificial insemination procedure (AI), and for 3-min observational periods before and after the procedure (averaged to create the baseline values shown here). Also shown are F and p values for the statistical test of this within-heifer comparison of the AI vs. baseline periods.
(DOCX)

S2 Table LS mean ± SE counts of ear position and body movement, separately by treatment. Naïve heifers were observed for 3 min during the artificial insemination procedure (AI), and for 3-min observational periods before and after the procedure (combined average shown for the non-AI baseline). Of the 12 heifers tested, 7 had previous positive reinforcement training (PRT) and 5 had not been trained. Values are shown separately for each combination of these two effects (i.e. AI vs. non—AI and PRT vs. control). Also shown are the F and p values for the statistical test of interaction between the within-heifer effect of AI vs. non-AI and the between-heifer effect of PRT.
(DOCX)
