# Supplementary material for: Behavioral responses to artificial insemination and the effect of positive reinforcement training
Source: PLoS One. 2024 Oct 10;19(10):e0310537. doi: 10.1371/journal.pone.0310537 (PMC11466390; doi:10.1371/journal.pone.0310537)
Supplement: S1 Table — Naïve heifers were observed for 3 min during the artificial insemination procedure (AI), and for 3-min observational periods before and after the procedure (averaged to create the baseline values shown here). Also shown are F and p values for the statistical test of this within-heifer comparison of the AI vs. baseline periods. (DOCX) [file pone.0310537.s001.docx]

**S1 Table: LS mean ± SE counts per observational period for each ear position and body movement assessed.**

|  | **Parameters** | **AI** | **Baseline** | **F_[1,10]_** | **p** |
| --- | --- | --- | --- | --- | --- |
| **Ear Positions** | Axial | 0.31 ± 0.82 | 4.57 ± 0.82 | 13.52 | 0.004 |
|  | Forward | 0.50 ± 0.51 | 3.45 ± 0.51 | 20.22 | 0.001 |
|  | Backward | 6.20 ± 0.97 | 6.03 ± 0.97 | 0.02 | 0.89 |
|  | Backward Pinned | 3.76 ± 0.76 | 0.12 ± 0.76 | 12.09 | 0.006 |
|  | Asymmetric | 0.96 ± 0.71 | 6.66 ± 0.71 | 32.30 | 0.0002 |
|  | Not Visible | 1.27 ± 0.83 | 3.67 ± 0.83 | 6.77 | 0.03 |
| **Movement Parameters** | Forward | 2.74 ± 0.62 | 1.74 ± 0.62 | 1.32 | 0.28 |
|  | Backward | 0.47 ± 0.41 | 1.42 ± 0.41 | 2.68 | 0.13 |
|  | Front Steps | 2.57 ± 0.74 | 2.56 ± 0.74 | 0.00 | 0.99 |
|  | Back Steps | 13.53 ± 2.91 | 11.17 ± 2.91 | 0.33 | 0.58 |

Naïve heifers were observed for 3 min during the artificial insemination procedure (AI), and for 3-min observational periods before and after the procedure (averaged to create the baseline values shown here). Also shown are F and p values for the statistical test of this within-heifer comparison of the AI vs. baseline periods.
