# Supplementary material for: Behavioral responses to artificial insemination and the effect of positive reinforcement training
Source: PLoS One. 2024 Oct 10;19(10):e0310537. doi: 10.1371/journal.pone.0310537 (PMC11466390; doi:10.1371/journal.pone.0310537)
Supplement: S2 Table — Naïve heifers were observed for 3 min during the artificial insemination procedure (AI), and for 3-min observational periods before and after the procedure (combined average shown for the non-AI baseline). Of the 12 heifers tested, 7 had previous positive reinforcement training (PRT) and 5 had not been trained. Values are shown separately for each combination of these two effects (i.e. AI vs. non—AI and PRT vs. control). Also shown are the F and p values for the statistical test of interaction between the within-heifer effect of AI vs. non-AI and the between-heifer effect of PRT. (DOCX) [file pone.0310537.s002.docx]

**S2 Table: LS mean ± SE counts of ear position and body movement, separately by treatment.**

| **Treatment* Period Interaction** | | **LS Means** | | | | **F_[1,10]_** | **P** |
| --- | --- | --- | --- | --- | --- | --- | --- |
|  |  | **AI/Control** | **AI/PRT** | **Baseline/Control** | **Baseline/PRT** |  |  |
| **Ear Positions** | Axial | 0.20 ± 1.25 | 0.43 ± 1.06 | 4.00 ± 1.26 | 5.14 ± 1.06 | 0.16 | 0.70 |
|  | Forward | 1.00 ± 0.78 | 0.00 ± 0.66 | 4.90 ± 0.78 | 2.00 ± 0.66 | 2.10 | 0.18 |
|  | Backward | 5.40 ± 1.47 | 7.00 ± 1.25 | 5.20 ± 1.47 | 7.00 ± 1.25 | 0.00 | 0.98 |
|  | Backward Pinned | 4.80 ± 1.16 | 2.71 ± 0.98 | 0.10 ± 1.16 | 0.14 ± 0.98 | 1.04 | 0.33 |
|  | Asymmetric | 1.20 ± 1.08 | 0.71 ± 0.92 | 7.10 ± 1.08 | 6.21 ± 0.92 | 0.04 | 0.85 |
|  | Not Visible | 0.40 ± 1.26 | 2.14 ± 1.07 | 3.70 ± 1.26 | 3.64 ± 1.07 | 0.95 | 0.35 |
| **Movement Parameters** | Forward | 4.20 ± 0.95 | 1.29 ± 0.80 | 2.90 ± 0.95 | 0.57 ± 0.80 | 0.11 | 0.75 |
|  | Backward | 0.80 ± 0.62 | 0.14 ± 0.53 | 2.20 ± 0.63 | 0.64 ± 0.53 | 0.60 | 0.46 |
|  | Front Steps | 4.00 ± 1.12 | 1.14 ± 0.95 | 3.70 ± 1.13 | 1.43 ± 0.95 | 0.08 | 0.78 |
|  | Back Steps | 15.20 ± 4.45 | 11.86 ± 3.76 | 9.70 ± 4.45 | 12.64 ± 3.75 | 0.58 | 0.50 |

Naïve heifers were observed for 3 min during the artificial insemination procedure (AI), and for 3-min observational periods before and after the procedure (combined average shown for the non-AI baseline). Of the 12 heifers tested, 7 had previous positive reinforcement training (PRT) and 5 had not been trained. Values are shown separately for each combination of these two effects (i.e. AI vs. non—AI and PRT vs. control). Also shown are the F and p values for the statistical test of interaction between the within-heifer effect AI vs. non-AI and the between-heifer effect of PRT.
